# Growth performance, carcass merit, and nitrogen status of long-fed feedlot steers in response to dietary protein concentrations: a comparison of industry-average and reduced protein diets

**DOI:** 10.1093/tas/txag037

**Published:** 2026-03-25

**Authors:** Alejandro M Pittaluga, Alejandro E Relling

**Affiliations:** Department of Animal Sciences, The Ohio State University, Wooster, OH 44691, United States; Department of Animal Sciences, The Ohio State University, Wooster, OH 44691, United States

**Keywords:** crude protein, growth performance, long-fed cattle, plasma urea nitrogen

## Abstract

Angus × SimAngus-crossbred steers (*n* = 93; body weight [BW] = 342 ± 29 kg) were used in a randomized complete block design to examine the effect of feeding finishing diets with crude protein (CP) contents similar or below industry averages on growth performance, carcass characteristics, and plasma urea nitrogen (PUN). Steers were blocked by BW and randomly assigned to 1 of 2 treatments (3 pens/treatment; 15 steers/pen). Treatments consisted of a dry rolled corn-based diet offered ad libitum during 206 d with either 11.5% or 13.5% CP (Dry matter [DM] basis). The experimental diets were formulated to be isoenergetic. Interim BW were recorded at 28 d-intervals and blood samples were collected from all steers on d 56, 112, and 197 for the analysis of PUN. All statistical analyses were conducted using the MIXED procedure of SAS 9.4 considering the pen as the experimental unit. No treatment × period interaction was detected for BW, average daily gain (ADG), dry matter intake (DMI), and gain to feed ratio (G: F; *P* ≥ 0.11). Steers administrated the diet containing 11.5% CP displayed greater DMI (*P* = 0.04) and tended to be heavier (*P* = 0.08) than their counterpart fed the diet with 13.5% CP. No treatment effect was evidenced for ADG and G: F (*P* ≥ 0.15). A treatment × period interaction was detected for relative DMI (% BW), where steers fed the diet with 11.5% CP showed a greater DMI during the early and mid-stages of the feeding period (*P* = 0.01). Steers fed the 11.5% CP diet had greater carcass-adjusted final BW and ADG than steers fed the 13.5% CP diet (*P* ≤ 0.01). A tendency for a treatment × d interaction was observed (*P* = 0.07) for PUN, with steers fed the 11.5% CP diet displaying lower PUN concentration in d 56 compared to those fed the 13.5% CP diet. In addition, the concentrations of PUN in d 197 were lower compared to those observed in d 56 and 112 (*P* < 0.01). Steers offered the diet with 11.5% CP had a heavier hot carcass weight and increased dressing percentage relative to steers fed the diet with 13.5% CP (*P* ≤ 0.02). The remaining carcass characteristics were unaffected by treatments (*P* ≥ 0.15). Taken together, results from this investigation demonstrate that finishing diets with 11.5% CP provides steers with sufficient protein, and although the feed intake relative to BW substantially declines during late-stage finishing, this decline does not impose protein-induced constraints on productivity.

## Teaser

Current trends of prolonged finishing periods might impact protein adequacy, warranting reevaluation of optimal crude protein supply. Our research explored how dietary protein contents similar or below industry averages affects productivity in feedlot cattle fed for 206 d.

## Introduction

Accurately estimating the necessary provision of dietary crude protein (CP) to satisfy the feedlot cattle requirements, necessitates consideration of the dynamic fluctuations in protein accretion rates and feed intake throughout the finishing phase. Initially, greater protein deposition rates increase protein demands, however as cattle approach maturity, the lower protein accretion rates along with greater feed intake diminishes dietary CP concentration requirements (Owens et al. 1995; Vasconcelos et al. 2007). In support of this tenet, applying phase-feeding strategies whereby concentrations of CP are decreased as days on feed (DOF) progress, had no detrimental effect on growth performance of feedlot cattle (Cole et al. 2006; Vasconcelos et al. 2006). Furthermore, withdrawing supplemental CP in late finishing stages may alleviate nitrogen excretion without compromising productivity (Preston 1980; May et al. 2014). However, on-farm adoption of precision protein feeding has been stymied by the lack of operational practicality of these management schemes. To maximize growth performance and minimize nitrogen wastage, maintaining constant concentrations of CP between 11.5 and 13% of the diet dry matter (DM) during the finishing stage has been suggested to be optimal (Vasconcelos et al. 2009). Nevertheless, the CP concentrations of commercial finishing diets usually exceeds the recommended range from the literature with an average of 13.4% as a consequence of the habitual inclusion of non-starch protein-rich corn byproducts as partial replacements of corn grain (Samuelson et al. 2016a; Crawford et al. 2022). While controversies about the sustainability of ruminant agriculture are regularly centered on methane emissions relative to meat and milk production, as ruminants increasingly draw public scrutiny, scientific evidence to justify protein overfeeding will be necessary for complying with concerns about nitrogen pollution and responsible environmental stewardship.

The feedlot industry’s shift towards extended finishing phases, aims to capitalize on increased carcass weights and mitigate the inflated feeder cattle costs (Sperber et al. 2024). However, as the feeding period lengthen and steers reach heavier, fatter endpoints, their absolute dry matter intake (DMI; kg/d) tends to plateau while BW continues to increase (Galyean et al. 2023; Martinez et al. 2023). Consequently, marked declines in DMI relative to BW occur, a phenomenon exacerbated by current predictive equations that overestimate feed intake, particularly toward the end of these prolonged periods (Pittaluga et al. 2025). This may create a divergence, where protein supply may become insufficient to cover the rising maintenance costs and the ongoing growth, potentially precipitating productivity constraints as a result of inadequate protein consumption. This scenario may justify the prevailing commercial practice of administering diets with CP contents above recommendations to mitigate eventual late-stage productivity losses. Moreover, the DMI relative to BW metric might serve as a proxy for such divergence between CP supply and increasing BW. Therefore, the objective of this experiment was to evaluate the effect of feeding finishing diets with CP contents similar or below industry standards on growth performance, carcass characteristics, and plasma urea nitrogen (PUN) concentrations in feedlot cattle fed for over 200 d. We hypothesized that lowering the concentrations of CP was going to decrease growth performance and carcass characteristics of feedlot steers.

## Materials and methods

All experimental procedures were approved by the Institutional Animal Care and Use committee of The Ohio State University (#2025A00000025) and followed the guidelines recommended in the Guide for the Care and Use of Agricultural Animal in Agricultural Research and Teaching (FASS 2010).

### Experimental design, treatments, and housing

The experiment was conducted at the Eastern Agricultural Research Station of The Ohio State University (Belle Valley, Noble County, OH). Angus × SimAngus-crossbred steers (*n* = 93; [BW] = 342 ± 29 kg) were used in a randomized complete block design to evaluate the effect of varying dietary CP concentrations on growth performance, carcass characteristics, and PUN in long-fed feedlot cattle. After weaning, steers were fed soybean hulls and grass hay for 40 d, and subsequently group fed a ration composed of 60% ground corn, 10% soybean meal, 28% soybean hulls, and 2% animal-vegetable blend fat, with ad libitum access to grass hay for 75 d. Steers were then transitioned to the experimental diets over a 24-d period by replacing grass hay with dry rolled corn in a step-wise fashion using 3 intermediate diets fed for 8 d each. Steers initial BW, calculated as the average of a 2-consecutive day BW recorded on d 0 and 1 (relative to treatment administration) was used as the blocking criteria. On d 1, steers were randomly selected to receive 1 of 2 dietary treatments (3 pens/treatment; 15–16 steers/pen) consisting of a dry rolled corn-based diet offered ad libitum during 206 d with a CP concentration of either 11.5% or 13.5% (DM basis; Table 1). Pens contained two individual animal feed intake monitoring systems (GrowSafe, GrowSafe Systems Ltd), which were filled once daily at 0900 h. Adaptation of the feeding systems was described previously (Pittaluga et al. 2021), with a diet that that was a 50:50 mixed of both experimental diets. The experimental diets were formulated to be isoenergetic and to exceed cattle requirements of vitamins and minerals for an estimated average daily gain (ADG) of 1.4 kg/d (NASEM 2016). On d 57, steers were implanted with Synovex ONE Feedlot (Zoetis, Parsippany-Troy Hills, NJ; 200 mg of trenbolone acetate and 28 mg of estradiol benzoate).

**Table 1:** Ingredients and analyzed nutrient content of the experimental diets fed to steers.

Item	11.5%CP	13.5%CP
**Ingredient, % of DM^1^**		
** DDGS^1^**	11.50	17.00
** Dry rolled corn**	68.50	63.00
** Soybean meal**	1.50	3.70
** Soybean hulls**	5.50	3.30
** Grass hay**	10.00	10.00
** Supplemental Premix^2^**	3.00	3.00
**Analyzed composition, % of DM**		
** CP^1^**	11.27	13.35
** NDF^1^**	19.65	20.40
** ADF^1^**	10.26	9.68
** EE^1^**	2.72	2.82
** Ash**	6.62	6.73
** RUP^1^**	6.81	7.78
** RDP^1^**	5.13	6.17
** NEm^3^**	1.98	1.98
** NEg^4^**	1.31	1.32

1 Abbreviations: DM, dry matter; DDGS, dry distillers grains with solubles; CP, crude protein; NDF, neutral detergent fiber; ADF, acid detergent fiber, EE, ether, Extractable; RUP, ruminally undegradable protein; RDP, ruminally degradable protein.

2 11.667% urea, 53.333% limestone, 27.507% NaCl, 1.170% Se, 0.200% CuSO_4_, 0.617% ZnSO_4_, 0.370% MnSO_4_, 0.003% CoCO_3_, 0.227% vitamin A-30, 0.227% vitamin D-3, 0.667% vitamin E, 0.003% EDDI, 0.677% Rumensin 90 (Elanco Animal Health, Greenfield, IN).

3 NEm: estimated net energy for maintenance. NEm (Mcal/kg) = 1.37 × ME - 0.138 × ME^2^ + 0.0105 × ME^3—^1.12 (NASEM, 2000).

4 NEg: estimated net energy for gain. NEg (Mcal/kg) = 1.42 × ME + 0.174 × ME^2^ + 0.0122 × ME^3—^1.65 (NASEM, 2000).

Steers were vaccinated with Ultrachoice 7 and Bovi-shield Gold FP5L5 (Pfizer Animal Health) at weaning, and received a booster vaccination 25 d later. Steers were group-housed in pens (7.3 × 37.2 m) that included an area covered by a metal roof (7.3 × 8.5 m) and an outside loafing area (7.3 × 28.6 m). The flooring material under the covered space was comprised of crushed, compacted limestone (screenings), and the outside loafing area was concrete. Pens were divided by a 1.5 m high wood fence with a 10 cm separation between rectangular rails.

### Data recording and sample collection

Interim BW were recorded at 28 d-intervals, and the final BW was calculated as the average BW recorded on the last two d of the experiment (d 205 and 206). Steers were weighed before the morning feeding and were not withheld from feed or water. On d 206, steers were harvested at a commercial abattoir (Cargill, Wyalusing, PA), and carcass data were provided by a United States Department of Agriculture (USDA) grader. Carcasses were chilled for 48 h at −4°C and subsequently ribbed between the 12^th^ and 13^th^ ribs to determine backfat thickness, marbling score, *Longissimus* muscle (LM) area, and yield grade (YG).

Feed samples were collected biweekly and immediately frozen at −20°C for further nutrient composition analysis. Equal portions of each feed ingredient were composited, oven-dried (24 h at 105°C), and ground through a Wiley mill (1 mm screen, Arthur H. Thomas, Philadelphia, PA). Ingredients were analyzed for NDF and ADF (Ankom Technology method 5 and 6, respectively; Ankom 200 Fiber Analyzer, Ankom Technology), and ether extract (Ankom Technology method 2). Total nitrogen was analyzed using a LECO TruMac N Nitrogen Determinator (LECO Corporation, St. Joseph, MI) according to AOAC method (AOAC 1997; #990.03). The content of CP was calculated as *N* × 6.25. The ash content was determined by ignition of samples at 600°C for 2 h using a Thermolyte muffle oven Model F30420C (Thermo Scientific, Waltham, MA) according to the AOAC method (AOAC 2005; #942.05). The net energy content of the diets was calculated from their analyzed content of total digestible nutrients ([TDN] % = 93.53 − 1.03 × % ADF, Undersander et al. [1993]) and metabolizable energy ([ME] Mcal/kg = TDN × 4.409 × 0.82) before the beginning of the trial following NASEM (2000) equations. Proportions of ruminally undegradable and degradable protein were calculated based on tabular values (NASEM 2016).

Blood samples were collected from all steers before the morning feed on d 56, 112, and 197 for the analysis of PUN. Blood was collected via jugular venipuncture into evacuated tubes containing 7.2 mg of disodium EDTA and immediately placed on ice. Upon arriving to the laboratory, plasma was obtained from blood samples after centrifugation for 10 min at 2000 × *g* and 4°C. Plasma samples were aliquoted into three individual 1.5 mL micro polypropylene tubes and stored at −80°C for later analysis. After thawing plasma samples, PUN was analyzed using a commercially available spectrophotometric assay (Catalog number K024-H1; Urea Nitrogen Detection Kit, Arbor Assays, Ann Arbor, MI) and by following manufacturer instructions. Briefly, plasma samples were diluted with distilled water (1:20) and 50 µL of the diluted samples and standards were mixed with Color Reagent A and Color Reagent B. Absorbance was measured at 450 nm after incubation for 30 min at room temperature. Sensitivity for urea was determined as 0.030 mg/dL. The intra- and inter-assay CV for the analyses of PUN were ≤ 11.5%.

### Statistical analyses

All statistical analyses were conducted using the MIXED procedure of SAS 9.4 (SAS Inst. Inc., Cary, NC). Growth performance and PUN were analyzed as repeated measures and considering the pen as the experimental unit. The models were fitted with individual animal data and included the fixed effects of treatment, time (day), and their interaction, as well as the random effect of the BW block and pen within the BW block. The first-order autoregressive covariance structure was used for the analysis, as having the lowest Akaike Information Criterion. For the cumulative growth performance and the carcass characteristics, the model included the fixed effect of treatment and the random effect of the BW block and pen within the BW block. For the variables with a *P*-value ≤ 0.10 for treatment by time interaction, the LS-means were separated using the PDIFF and SLICE option of SAS. Differences were set at *P*-value ≤ 0.05; and tendencies at *P*-value > 0.05 and *P*-value ≤ 0.10.

## Results

**Figure 1:**
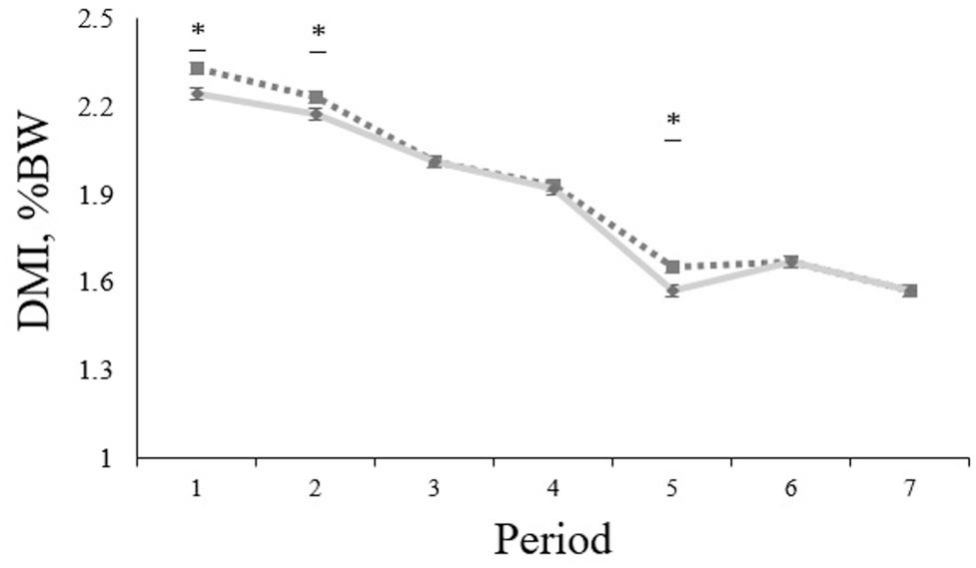
Mean ± SEM (±0.02) of dry matter intake (DMI) expressed as a % of the body weight (BW) in feedlot steers fed diets with 11.5% CP (closed square) or 13.5% CP (closed diamond). Period 1, 2, 3, 4, 5, 6, and 7 represent the DMI of steers recorded from d 1–28 (period 1), 29–56 (period 2), 57–84 (period 3), 85–112 (period 4), 113–140 (period 5), 141–168 (period 6), and 169–206 (period 7). A tendency for a treatment × d interaction was evidenced (*P* = 0.07), wherein steers fed diets with 11.5% CP showed a greater DMI than steers fed diets with 13.5% CP in periods 1, 2, and 5. Symbols above points indicate differences (*P* ≤ 0.05*) between treatments at each time point.

Although the actual CP concentrations of the experimental diets were 11.27 and 13.35% (DM basis) for the 11.5 and 13.5% treatments, respectively (Table 1), the formulated CP values will continue to be used as the treatment identifiers. On growth performance parameters, no treatment × period interactions were detected for BW, ADG, DMI, or gain to feed (G: F) ratio (*P* ≥ 0.11; Table 2). However, a treatment effect (*P* = 0.04) and a tendency for a treatment effect (*P* = 0.08) were observed for DMI and BW, respectively. Steers administrated the diet containing 11.5% CP exhibited a greater DMI and were marginally heavier than their cohort fed the diet with 13.5% CP. No treatment effect was evidenced for ADG and G: F (*P* ≥ 0.15). When expressing DMI as a percentage of BW, a treatment × period interaction was detected (*P* = 0.01), with steers fed the 11.5% CP diet showing greater DMI than steers fed the 13.5% CP diet at the beginning of the experiment (d 1 to 56) and from d 113 to 140 (Fig. 1). A time effect was evidenced for BW, ADG, DMI, and G: F (*P* ≤ 0.01); however, they are outside the scope of this study and will therefore not be discussed. Cumulatively, steers fed the 11.5% CP diet exhibited a marginally greater DMI (*P* = 0.10) and increased carcass-adjusted final BW and ADG (*P* ≤ 0.01) compared to those fed the 13.5% CP diet (Table 3).

**Table 2:** Effect of varying concentrations of dietary crude protein on growth performance of feedlot finishing steers.

	Treatment^1^			*P*-value^3^		
Item	11.5%CP	13.5%CP	SEM^2^	Trt	*P*	Trt × *P*
**n**	47	46				
**BW^4^, kg**			24	0.08	<0.01	0.20
** IBW^5^**	329	327				
** 28 d**	389	385				
** 56 d**	430	425				
** 84 d**	486	477				
** 112 d**	532	523				
** 140 d**	574	563				
** 168 d**	600	590				
** 206 d**	657	648				
**ADG^6^, kg/d**			0.08	0.15	<0.01	0.63
** 1 to 28 d**	2.07	1.98				
** 29 to 56 d**	1.57	1.54				
** 57 to 84 d**	1.99	1.84				
** 85 to 112 d**	1.41	1.45				
** 113 to 140 d**	1.68	1.57				
** 141 to 168 d**	1.32	1.34				
** 169 to 206**	1.37	1.40				
**DMI^7^, kg/d**			0.47	0.04	<0.01	0.11
** 1 to 28 d**	9.47	9.00				
** 29 to 56 d**	9.99	9.64				
** 57 to 84 d**	10.19	9.99				
** 85 to 112 d**	10.73	10.49				
** 113 to 140 d**	9.90	9.22				
** 141 to 168 d**	10.48	10.28				
** 169 to 206 d**	10.73	10.61				
**G: F^8^**			0.006	0.52	<0.01	0.73
** 1 to 28 d**	0.220	0.222				
** 29 to 56 d**	0.157	0.161				
** 57 to 84 d**	0.196	0.184				
** 85 to 112 d**	0.132	0.140				
** 113 to 140 d**	0.170	0.167				
** 141 to 168 d**	0.124	0.132				
** 169 to 206 d**	0.128	0.132				

a–b Within a row, means without a common superscript differ (*P* ≤ 0.05).

Significance was declared at *P* ≤ 0.05; and tendencies were declared at *P* > 0.05 and *P* ≤ 0.10.

1 11.5%CP = diet formulated to contain 11.5% of CP (DM basis); 13.5%CP = diet formulated to contain 13.5% of CP (DM basis).

2 Pooled standard error of treatments means.

3 Trt = treatment; *P* = period; Trt × *P* = treatment by period interaction.

4 A 4% pencil shrink was applied to all BW measures.

5 Initial body weight.

6 Average daily gain.

7 Dry matter intake.

8 Gain to feed ratio.

**Table 3:** Effect of varying concentrations of dietary crude protein on live and carcass-adjusted cumulative growth performance of feedlot finishing steers.

	Treatment^1^			
Item	11.5%CP	13.5%CP	SEM^2^	*P*-value
**n**	47	46		
**IBW^3^, kg**	329	327	22	0.72
**FBW^4^, kg**	657	648	22	0.08
**Carcass-adjusted FBW^5^, kg**	665	651	4	0.01
**DMI^6^, kg/d**	10.31	10.04	0.42	0.10
**Live basis**				
** ADG^7^, kg/d**	1.58	1.55	0.05	0.28
** G: F^8^**	0.154	0.155	0.002	0.68
**Carcass-adjusted (HCW/0.62)^9^**				
** ADG^7^, kg/d**	1.64	1.56	0.05	<0.01
** G: F^8^**	0.152	0.150	0.002	0.35

Significance was declared at *P* ≤ 0.05; and tendencies were declared at *P* > 0.05 and *P* ≤ 0.10.

1 11.5%CP = diet formulated to contain 11.5% of CP (DM basis); 13.5%CP = diet formulated to contain 13.5% of CP (DM basis).

2 Pooled standard error of treatments means.

3 Initial BW with a 4% pencil shrink applied.

4 Final BW recorded on d 206 with a 4% pencil shrink applied.

5 Final BW calculated by dividing hot carcass weight by a common dressing of 62.0%.

6 Dry matter intake.

7 Average daily gain.

8 Gain to feed.

9 Hot carcass weight adjusted to a common dressing percentage of 62.0%.

**Figure 2:**
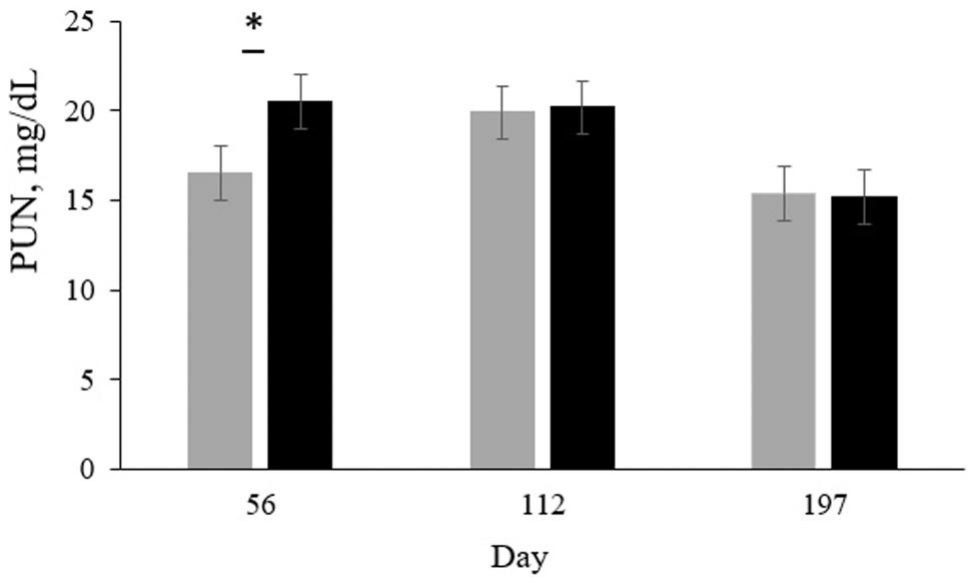
Mean ± SEM of plasma urea nitrogen (PUN) of feedlot steers fed diets with 11.5% CP (grey) or 13.5% CP (black). A tendency for a treatment × d interaction was detected (*P* = 0.07) for PUN, where steers fed diets with 11.5% CP exhibited a lower concentration of PUN than steers fed diets with 13.5% CP in d 56. In d 112 and 197, the concentration of PUN did not differ between treatments. In addition, a d effect was observed (*P* < 0.01), where concentrations of PUN in d 197 were lower compared to those observed in d 56 and 112.

A tendency for a treatment × d interaction was observed (*P* = 0.07; Fig. 2) for PUN, with steers fed the 11.5% CP diet displaying lower PUN concentration in d 56 compared to those fed the 13.5% CP diet. In d 112 and 197, the concentrations of PUN did not differ between treatments. In addition, a d effect was observed (*P* < 0.01), where concentrations of PUN in d 197 were lower compared to those observed in d 56 and 112. The mean (±SEM) PUN in d 56, 112, and 197 was 18.5, 20.1, and 15.3 mg/dL, respectively (±1.1).

For the carcass characteristics, steers fed the diet with 11.5% CP had a heavier hot carcass weight (HCW) and dressing percentage relative to steers fed the diet with 13.5% CP (*P* ≤ 0.02; Table 4). No other treatment effect was detected for the remaining carcass characteristics (*P* ≥ 0.15), ie 12^th^ rib fat thickness, *Longissimus* muscle area, marbling score, yield grade, and empty body fat.

**Table 4:** Effect of varying concentrations of dietary crude protein on the carcass characteristics of feedlot finishing steers.

	Treatment^1^			
Item	11.5%CP	13.5%CP	SEM^2^	*P*-value
**n**	47	46		
**Dressing, %**	62.94	62.15	0.22	0.02
**HCW^3^, Kg**	413	403	4	0.01
**LM area, cm^2^**	93.1	90.1	2.2	0.15
**12^th^ rib fat, cm**	1.88	1.84	0.04	0.46
**MS^4^**	671	663	16	0.73
**YG^5^**	4.1	4.1	0.1	0.69
**EBF^6^**	33.44	33.17	0.34	0.53

Significance was declared at *P* ≤ 0.05; and tendencies were declared at *P* > 0.05 and *P* ≤ 0.10.

1 11.5%CP = diet formulated to contain 11.5% of CP (DM basis); 13.5%CP = diet formulated to contain 13.5% of CP (DM basis).

2 Pooled standard error of treatments means.

3 Hot carcass weight.

4 Marbling score; scale: 400–490 = slight, 500–590 = small, 600–690 = modest, 700–790 = moderate, 800–890 = slightly abundant.

5 Yield grade.

6 EBF = empty body fat; EBF = 17.76207 + (4.68142 × 12^th^ rib fat) + (0.01945 × HCW) + (0.81855 × QG) − (0.06754 × LM area), (Guiroy et al. 2001).

## Discusion

The concept of optimal CP concentrations in feedlot finishing diets aims to maximize nitrogen retention in animal tissues while minimizing manure nitrogen output. In this sense, protein precision feeding, which involves adjusting dietary CP supply to match the decreasing protein demands of feedlot cattle throughout the finishing phase, seems the approach most congruent with CP feeding optimization (Erickson and Klopfenstein 2001; Vasconcelos et al. 2006). However, maintaining a constant CP concentration during the finishing phase offers a more applicable scheme considering within-pen variations in cattle requirements and the logistical challenges of tailoring protein inclusion to the decreasing protein needs of mature cattle (Vasconcelos et al. 2007; Samuelson et al. 2023). To satisfy metabolizable protein requirements in feedlot cattle, current recommendations advocate for a dietary CP range of 11.5% to 13.0% (DM basis), depending on the net energy of the diet and the DMI of cattle (NASEM 2016). Formulating diets below this threshold may enhance nitrogen retention, yet frequently at the expense of maximal growth potential (Cole et al. 2006). Furthermore, providing CP in excess of requirements fails to elicit a productive response, implying that growth performance is not a function of protein intake once physiological requirements are surpassed (Gleghorn et al. 2004; Vasconcelos et al. 2009). Nonetheless, the increasing utilization of protein-rich co-products derived from wet- and dry-milling processes of corn grain, has led to commercial finishing diets exceeding recommended CP concentrations, with an average of 13.4% and a maximum of 15.9% (DM basis) reported in recent surveys (Samuelson et al. 2016a). As environmental concerns and public scrutiny of ruminant production intensify, scientific justification for routine nitrogen overfeeding and associated pollutant emissions will become increasingly necessary. Moreover, nitrogen excretion due to protein overfeeding is projected to be amplified as the feedlot industry continues extending the finishing phase to capture additional revenue from heavier carcasses. Feeding high-protein diets to mature cattle with decreasing protein requirements for longer periods will likely exacerbate the oversupply of dietary nitrogen. However, prominent declines in feed intake relative to BW during late-stage finishing may constrain growth performance due to protein inadequacy. More specifically, as the feeding period lengthen, absolute DMI plateaus while BW continues to increase (Galyean et al. 2023; Martinez et al. 2023). This may create a divergence, where protein supply may become insufficient to cover the rising maintenance costs and the ongoing growth. This scenario may justify the prevailing commercial practice of administering diets with CP contents above recommendations to mitigate eventual late-stage productivity losses.

Commonly employed predictive equations overestimated the DMI of late-stage feedlot steers relative to the observed DMI, corroborating Pittaluga et al. (2025) results that a quadratic model for BW provides a more robust fit to the data than the linear model of BW reported previously (Anele et al. 2014; NASEM 2016, Fig. 3). Contrary to our hypothesis, despite a marked decline in the feed intake relative to BW in the terminal segments of the finishing stage, the absence of growth performance improvements in steers fed the 13.5% CP diet suggests that the DMI decrements were insufficient to induce productivity limitations as a function of protein inadequacy. Notably, steers fed the diet containing 11.5% CP exhibited greater DMI throughout the experimental period and transient increases in DMI relative to BW, culminating in greater carcass-adjusted ADG, and heavier BW and HCW compared to steers fed the diet with 13.5% CP. This observation is consonant with previous studies indicating that elevated dietary protein concentrations might cause reductions in feed intake. For instance, Hales et al. (2016) reported that increasing the concentration of protein in the diet of feedlot steers from 13.5 to 17.5% reduced DMI without influencing ADG, while Samuelson et al. (2016b) observed DMI declines concomitant with linear decreases in ADG when CP concentrations were increased from 13.5 to 15.8%. However, these findings are at dissidence with the meta-analysis by Owens et al. (2014), which shows that increasing dietary CP tends to augment DMI. In addition, recent trials have reported unchanged DMI and ADG when feeding feedlot diets with CP contents exceeding industry standards, although, increasing ruminally degradable protein (RDP) proportions proved beneficial for live and carcass-adjusted growth performance (Samuelson et al. 2023; Ross et al. 2024). Despite the inherent inter-study heterogeneity in diet composition and experimental conditions leading to discordant outcomes, feed consumption and growth performance are generally not expected to decrease from feeding diets with a protein content above current industry standards.

**Figure 3:**
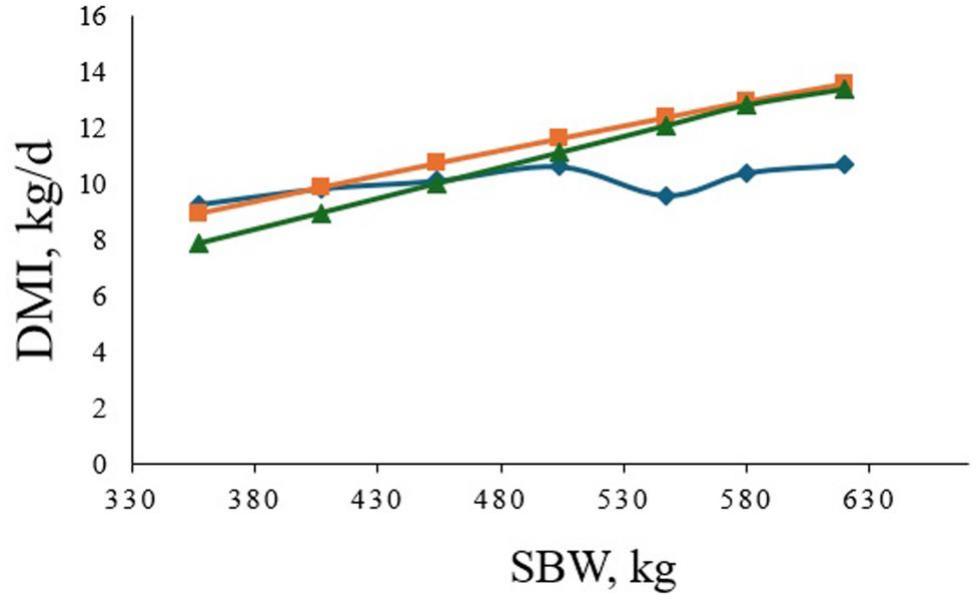
Comparison of the observed dry matter intake (DMI) of all feedlot steers from the experiment (blue diamond) with the Anele et al. (2014; green triangle) and NASEM (2016; orange square) equations for predicting DMI. The observed DMI portrays the average DMI recorded from d 1–28 (period 1), 29–56 (period 2), 57–84 (period 3), 85–112 (period 4), 113–140 (period 5), 141–168 (period 6), and 169–206 (period 7) as a function of the average shrunk BW (initial + final shrunk BW divided by 2) of each period. For the Anele et al. (2014) equation, DMI, kg/d = (1.2425 + 1.9218 × NEm—0.7259 × NEm^2^) × BW, and for the NASEM (2016) equation, DMI, kg/d = [BW^0.75^ × (0.2453 × NEm—0.0466 × NEm^2—^0.0869)]/NEm. NEm = dietary NEm concentration (Mcal/kg of DM) and BW (kg) = average shrunk BW (initial + final shrunk BW divided by 2). Results show that, as feedlot steers get heavier (>500 kg SBW), current predictive equations overpredict DMI.

In the present study, CP concentrations were increased primarily by substituting dry-rolled corn with distillers’ grains, which may have contributed to growth performance and DMI depressions given its elevated content of fat, elemental sulfur, and sulfuric acid (May et al. 2010; Luebbe et al. 2012; Pittaluga et al. 2021). However, the use of de-oiled distillers’ grains and the relatively small difference in distillers’ grains content between the 11.5% and the 13.5% CP diets likely preclude the nutrient profile of distillers grains as the primary driver of DMI reductions.

Following absorption into the portal circulation, excess nitrogen from the diet (NH_3_ and amino acids) is converted to urea through a detoxifying bicompartmental cycle in hepatocytes. Evidence indicates NH_3_ detoxification imposes a metabolic cost, augmenting NEm requirements (Reynolds, 1992) and diminishing growth performance. Concretely, up to 6% increases in NEm requirements of growing cattle were observed by Jennings et al. (2018) when increasing the CP concentration of the diet from 13.8 to 19.5%. Yet, this putative energetic burden on cattle metabolism necessitates further verification. Regardless of these considerations, the exact causal factors underlying the observed results from this study remain to be elucidated.

The analysis of PUN in cattle serves as a tool to gauge the nitrogen status or utilization efficiency, correlating with urinary nitrogen excretion. For feedlot cattle, a PUN threshold of 9 mg/dL has been suggested as indicative of nitrogen wastage (Cole et al. 2003; Vasconcelos et al. 2009). As dietary protein increases and the finishing phase advances, PUN usually increases, intensifying nitrogen wastage (Vasconcelos et al. 2006; Bailey et al. 2008; Ross et al. 2024). In the current experiment, feeding 13.5% CP diets resulted in transient PUN increases relative to steer fed 11.5% CP diets at the beginning of the feeding period, with values equalizing thereafter. Notably, PUN values for both treatments were substantially above the 9 mg/dL threshold throughout the experiment. Intriguingly, PUN values decreased by the end of the experiment, contradicting the projected trend. This observations partially aligns with previous trials where serum urea nitrogen concentrations remained unmodified with the progression of the finishing phase (Gleghorn et al. 2004; Cole et al. 2006). While differences in postprandial time of sampling and RDP ingestion could plausibly explain discrepancies in blood urea nitrogen (Cole et al. 2006), the precise contributory causes, however, remain elusive.

## Conclusions

Administrating diets with 11.5% CP compared with 13.5% CP concentration enhanced feed consumption and led to transient increases in feed intake relative to BW, resulting in heavier BW and carcasses compared to diets simulating industry standards. Despite a marked decline in feed intake relative to BW in late finishing stages occurred, decrements were insufficient to impose protein-induced constraints on productivity. Growth performance and PUN values evidenced throughout the experiment suggest that 11.5% CP diets fulfill cattle requirements of dietary protein. Feature research focused on unveiling the extent to which nitrogen ingestion can be reduced and quantifying the emissions of nitrogenous gases from manure relative to production traits will be advantageous for complying with responsible environmental stewardship.

## List of abbreviations

ADF: acid detergent fiber
ADG: average daily gain
BW: body weight
CP: crude protein
DM: dry matter
DMI: dry matter intake
G: F: gain to feed ratio
HCW: hot carcass weight
MP: metabolizable protein
NDF: neutral detergent fiber
PUN: plasma urea nitrogen
RDP: ruminally degradable protein
RUP: ruminally undegradable protein
