# Correction to: Growth performance, carcass merit, and nitrogen status of long-fed feedlot steers in response to dietary protein concentrations: a comparison of industry-average and reduced protein diets

**DOI:** 10.1093/tas/txag080

**Published:** 2026-06-27

This is a correction to: Alejandro M Pittaluga, Alejandro E Relling, Growth performance, carcass merit, and nitrogen status of long-fed feedlot steers in response to dietary protein concentrations: a comparison of industry-average and reduced protein diets, *Translational Animal Science*, Volume 10, 2026, txag037, https://doi.org/10.1093/tas/txag037

In the originally published version of this manuscript, the title was incorrectly listed as **‘**Crude protein concentration in feedlot cattle growth performance, carcass merit, and nitrogen status of long-fed feedlot steers in response to dietary protein concentrations: a comparison of industry-average and reduced protein diets’.

The Publisher apologizes for this error, which has now been corrected.

The title has been updated in the published article to read ‘Growth performance, carcass merit, and nitrogen status of long-fed feedlot steers in response to dietary protein concentrations: a comparison of industry-average and reduced protein diets’.

